# Editorial: Modeling of Visual Cognition, Body Sense, Motor Control and Their Integrations

**DOI:** 10.3389/fncom.2016.00142

**Published:** 2016-12-27

**Authors:** Hong Qiao, Li Hu

**Affiliations:** ^1^State Key Lab of Management and Control for Complex Systems, Institute of Automation, Chinese Academy of SciencesBeijing, China; ^2^Chinese Academy of Sciences Center for Excellence in Brain Science and Intelligence TechnologyShanghai, China; ^3^University of Chinese Academy of SciencesBeijing, China; ^4^CAS Key Laboratory of Mental Health, Institute of Psychology (CAS)Beijing, China

**Keywords:** computational modeling, biological mechanism, visual cortex, pain prediction, bio-inspired model, machine learning, neural networks

The interdisciplinary studies between neuroscience and computer science have greatly promoted the development of these two fields. The achievements of these studies can help humans understand the essence of biological systems; provide computational platforms and intelligent methods for biological experiments; and improve the intelligence and performance of the algorithms in computer science.

We present 10 papers in this research topic, which are mainly focused on neural mechanisms underlying the perception of vision, motor and pain; computational modeling of visual processing; bio-inspired visual models; and novel machine learning algorithms to reliably predict pain.

## 1. Neural mechanisms research of vision and motor

As our dominant sense, the ultimate purpose of visual processing is to support us in perception, cognition, learning, and activities.

One article (Perry et al.) gives a brief review of alterations in visual processing near hand, which supports the hypothesis that there exist parallel, and separate, effector-based attentional systems. Whereas the oculomotor system enhances visual responses through gain modulation, and near-hand attention system sharpens features (such as orientation) relevant to reaching and grasping. This article provides a potential structure for visual-motor interaction modeling in bio-inspired imitation learning.

## 2. Computational modeling of visual processing

The work of Galeazzi et al. is much related to the review paper (Perry et al.) on visual processing near hand. The authors analyzed the functions of neurons in VisNet model through a biologically plausible process of unsupervised competitive learning and self-organization both with realistic and natural images. The experiments showed that individual output cells of the network could develop single, localized, hand-centered receptive fields which are invariant to retinal location. Eguchi et al. modified VisNet to model the neural representation of object shape in the primate ventral visual system. By unsupervised visually-guided learning, the individual neurons show similar firing properties with V4 and TEO. The neurons in the higher layer of the network could learn to respond to localized boundary contour elements and show translation invariance across different retinal locations through the use of a trace learning rule.

Both of these two computational modeling methods simulate the principles and mechanisms of the visual pathway, and may inspire the future work in bio-inspired visual modeling for image processing applications.

## 3. Bio-inspired visual models

Li et al. proposed an enhanced HMAX model for image categorization. By mimicking the attention modulation, memory processing and feature encoding mechanisms of visual cognition, a bottom-up saliency map, an unsupervised iterative clustering method and multi-feature fusion method are introduced to the HMAX model. The enhanced bio-inspired model with small memory size showed better accuracy than other unsupervised feature learning methods in Caltech101 dataset. Fu et al. proposed an CNN model for feature construction in text analysis. By modifying the CNN model to adapt to the text inputs, introducing similarity of asker-answer information as attention modulation, and bringing in reputation information to imitate memory, the improved CNN model showed better performance in answer recommendation task.

Different from the computational modeling of visual processing (Galeazzi et al.; Eguchi et al.), these two bio-inspired visual models had excellent performance in public datasets focusing on computer science application, which shows that biological research can promote the development of computer science.

## 4. Neural mechanisms underlying the perception of pain

Pain is a subjective first-person experience, and self-report is the gold standard to determine pain in various clinical practice. Considering that self-report of pain is not available in some vulnerable populations, the development of an objective assessment of pain would be highly needed in clinical applications (Huang et al., 2013). To achieve this aim, we need to (1) identify neural activity that could serve as a cortical signature for pain perception in humans using non-invasive functional neuroimaging techniques, e.g., electroencephalography (EEG) and functional magnetic resonance imaging (fMRI), and (2) develop novel algorithms that could reliably predict the perceived pain based on the identified pain-related neural responses (Hu and Iannetti, 2016).

Three articles provide recent advances to better understand the neural mechanism related to the central processing of pain perception. Guo et al. investigated the vigilance states of the brain when the subjects were suffering from acute pain or chronic pain, and demonstrated that the vigilance level to external sensory stimuli would be increased with acute pain, but decreased with chronic pain. These observations indicated that the study of pain-induced influences on cortical processing of non-nociceptive sensory information would be a doable way to differentiate acute pain and chronic pain, thus help monitor the progress of pain chronification in clinical practice (Guo et al.). In addition, Li et al. investigated the effects of placebo analgesia on spontaneous brain oscillations during tonic muscle pain. They observed that placebo-induced decreases in the subjective pain perception significantly correlated with the increases of the amplitude of alpha oscillations, which suggested that alpha oscillations in frontal-central region could serve as the cortical indicator of placebo effect on tonic muscle pain (Li et al.). Finally, Peng and Tang provided a comprehensive summary of the functional properties of pain-induced modulations of ongoing cortical oscillations. In addition to the traditional methods, they proposed that novel approaches should be adopted to comprehensively explore the dynamics of oscillatory activities associated with pain perception and behavior. Based on these understandings, Peng and Tang pointed out the potential clinical applications of neurostimuation techniques (e.g., repeated transcranial magnetic stimulation (rTMS) and transcranial alternating current stimulation (tACS)) based on the modulation of pain-related cortical oscillations, which could help promote the establishment of rational therapeutic strategy in the framework of intelligent systems.

## 5. Machine learning algorithms for pain prediction

Two articles in this Research Topic developed novel techniques to improve the performance of pain prediction based on non-invasive functional neuroimaging signals. Bai et al. observed that pain-evoked EEG responses were significantly correlated with spontaneous EEG activities at interindividual level, and proposed a normalization approach to reduce the interindividual variability of pain-evoked EEG responses based on the spontaneous EEG activities for each subject. In addition, Bai et al. found that the relationship between pain-evoked EEG responses and pain perception was nonlinear, which inspired them to develop a novel two-stage pain prediction strategy, a binary classification of low-pain and high-pain trials followed by a continuous prediction of high-pain trials to significantly improve the prediction accuracy (Bai et al.). From a different aspect, Tu et al. provided evidences showing that the joint use of both pre-stimulus ongoing and post-stimulus evoked EEG/fMRI activities could significantly improve the performance of pain prediction compared to using just post-stimulus evoked brain responses. Both studies (Bai et al.; Tu et al.) shed new lights on the development of novel algorithms that could improve the prediction accuracy based on functional neuroimaging signals.

Taken together, this research topic provides a series of work in the interdisciplinary studies of vision, motor and pain. The biological findings and models of the topic could inspired the future studies both in biology and computer science.

## Author contributions

HQ and LH are the organizers of the research topic “Editorial: Modeling of Visual Cognition, Body Sense, Motor Control and Their Integrations.” For this editorial, we discussed and built the outline together. HQ was in charge of Section 1, 2 and 3 and the figure. LH was in charge of Section 4, 5. Both of the authors commented on the manuscript.

## Funding

HQ was supported by the National Natural Science Foundation of China (No. 61210009, 61627808), the Beijing Municipal Science and Technology Commission (D16110400140000, D161100001416001), and the Strategic Priority Research Program of the CAS (No. XDB02080003). LH was supported by the National Natural Science Foundation of China (No. 31471082, 31671141) and the Scientific Foundation project of Institute of Psychology, Chinese Academy of Sciences (No. Y6CX021008). The funders had no role in study design, decision to publish, or preparation of the manuscript. The authors have declared that no competing interests exist.

### Conflict of interest statement

The authors declare that the research was conducted in the absence of any commercial or financial relationships that could be construed as a potential conflict of interest.
